# Fluorodeoxyglucose and ^11^C-Choline positron emission tomography for distinction of metastatic plexopathy and neuritis: a case report

**DOI:** 10.1186/1757-1626-2-9323

**Published:** 2009-12-15

**Authors:** Anna L Bartels, Clark J Zeebregts, Roelien H Enting, Riemer HJA Slart

**Affiliations:** 1 Departments of Neurology, University Medical Center Groningen, Groningen, The Netherlands; 2 Surgery (Division of Vascular Surgery), University Medical Center Groningen, Groningen, The Netherlands; 3 Nuclear Medicine and Molecular Imaging, University Medical Center Groningen, Groningen, The Netherlands

## Abstract

**Introduction:**

Fluorodeoxyglucose positron emission tomography scanning has an established role in the diagnostic work-up of many malignant diseases and also in the evaluation of cancer treatment response. Fluorodeoxyglucose positron emission tomography may, however be non-specific as infectious processes are depicted as well.

**Case presentation:**

We present a patient with longstanding leg pain and weakness due to plexopathy developed a few years after treatment for prostate cancer. Prostate-specific antigen was raised and magnetic resonance imaging showed contrast uptake in thickened sacral nerves, suspicious for metastasis. While fluorodeoxyglucose positron emission tomography showed increased uptake in the plexus region, ^11^C-Choline- positron emission tomography did not show any uptake. It was concluded that the FDG uptake reflected plexus neuritis and no tumor. Treatment for pain relief was started.

**Conclusion:**

^11^C-Choline- positron emission tomography can be used to detect metastasis in patients with plexopathy suspicious for malignancy, while fluorodeoxyglucose positron emission tomography is more sensitive to inflammatory processes.

## Introduction

Malignancy can affect the lumbosacral plexus in three ways: first, by direct spread of the primary tumor to envelop the plexus; second, by metastasis to surrounding soft and bony tissues; and third, by deposits to the plexus itself [1]. Direct infiltration to nerves is characterized by neuropathic pain with long duration of symptoms, during which only nerve enlargement is seen without signs of other metastatic disease [2]. Neuropathic pain can develop as a result of neuritis. Contrast enhancement on MRI can be seen in an affected plexus, however non-specific contrast imaging alone cannot differentiate between malignancy and other conditions. Recent advantages in positron emission tomography (PET) may differentiate better in difficult cases. We present a patient with plexopathy and a history of prostate carcinoma, in which ^11^C-Choline PET differentiated plexus neuritis from malignant plexopathy.

## Case presentation

A 82-year-old Caucasian man from Dutch presented with progressive pain in his right buttock since two years. The pain was most severe when sitting or lying, which also hindered his sleep. The pain radiated from the right buttock via the lateral leg to the third to fifth toes, and was described as stabbing pain. The pain now also progressed to his dorsal right foot and medial ankle. Pain did not increase with coughing or other pressure raising factors. He had a cold feeling in the leg and foot, and a numb feeling at the fore-medial lower leg. Furthermore, he developed loss of force of the right leg and used a walking aid since one year. In 2002, he was diagnosed with medium-differentiated prostate adenocarcinoma, which was treated with trans-urethral surgery and hormonal therapy. Later, orchidectomy was done to withdraw hormonal therapy. He had a catheter à demeure. His serum prostate-specific antigen (PSA) had risen since 2005 to 6.0 ng/L.

Neurological investigation showed a global paresis grade 4 of the right upper leg with a distal paralysis of the right gastrocnemius and peroneus muscles. His right leg was kept preferably in flexed hip and knee position. Achilles tendon reflex was absent in the right leg with low knee tendon reflex; other reflexes were normal. Sensibility was disturbed in the right distal leg, showing decreased position sense of the right leg and decreased pain sensation at the medial lower leg. Lasegue test gave pain in the right upper leg and calf muscle at 30°. There were no other neurological abnormalities. EMG showed sacral plexopathy with neurogenic myopathy of L5 and S1 innervated muscles. Lumbar punction did not show malignant cells or signs of infectious diseases. Differential diagnosis included neuritis or metastasis of the prostate carcinoma with perineural tumor growth.

**Figure 1:**
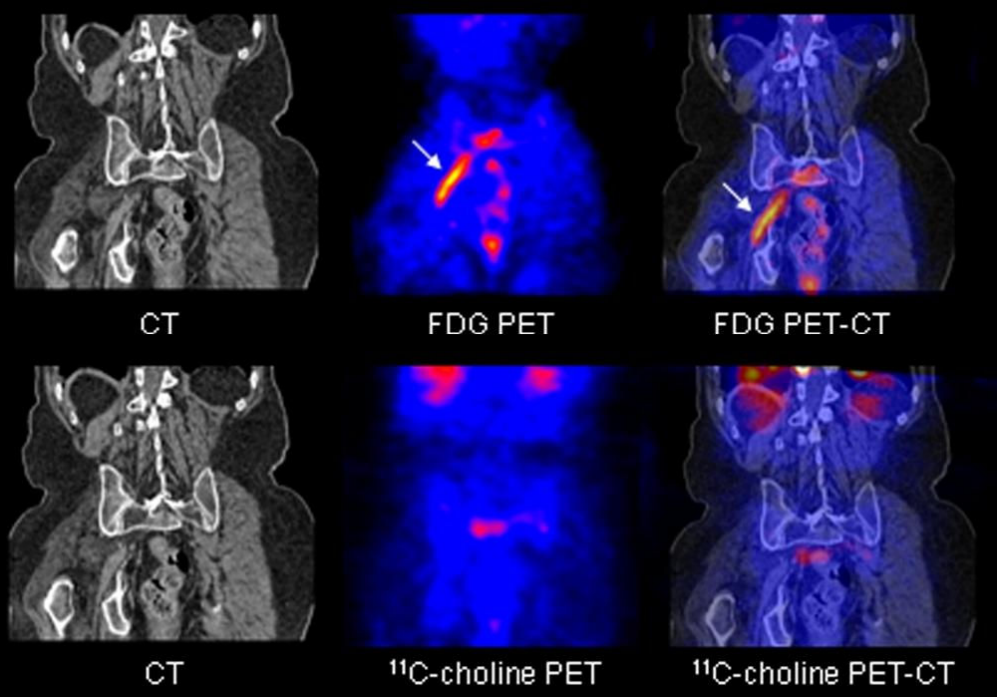
**Upper row shows increased FDG uptake in the right sacral plexus (arrow) on the fused FDG PET and fused FDG PET-CT scan**. Lower row shows normal ^11^C-choline distribution in the right sacral plexus region.

Contrast-enhanced CT scan of the abdomen/thorax showed a large prostate and no enlarged lymph nodes. At the right side, hydronephrosis and a dilated ureter were seen. Radius S1 and S2 at the right side showed diffuse thickening. There was atrophy of the gluteus maximus and medius muscles. Degenerative changes of thoracal and lumbar spine were seen. Bone scintigraphy was negative. MRI confirmed these findings and showed contrast enhancement of the right S1 and S2. Several constrictions were seen in the right ureter, but no obstructing metastasis was seen. FDG-PET was performed and showed increased uptake in the right sacral plexus, with a standardized uptake volume (SUV) of 2.3, and urine retention in the right pyelum and ureter (fig. 1). The increased FDG uptake in the sacral region, however, could not differentiate between plexus neuritis and metastatic plexopathy, although no tumor was seen. An additional PET scan was made with ^11^C-Choline, which showed no increased uptake in the sacral plexus (fig. 1).

As no other-infectious or malignant- causes were found, it was concluded that the plexopathy was most probably due to idiopathic chronic neuritis [3]. He was treated with pregabaline and ischiadic nerve blocks with bupivacaïne/kenacort for pain relief, which had reduced his pain complaints considerably after a few months. Follow-up after one year and eight months showed an unchanged neurological situation. His PSA was still slightly raised to 6.3 μg/L, which will be followed up by the urologist.

## Discussion

Our patient presented with longstanding neuropathic, radicular pain with a sacral plexopathy, which was suspicious for malignant plexopathy due to prostate carcinoma. MRI showed thickened sacral nerves with contrast enhancement, which could not exclude malignant plexopathy. Perineural tumor invasion and spread may occur in approximately 15% of prostate carcinoma and is associated with increased frequency of occult high-grade disease [4]. ^11^C-Choline is a biomarker for imaging prostate carcinoma, and is shown to be more accurate in determining distant prostate metastasis than conventional imaging methods [5,6]. De Jong et al. reported sensitivity, specificity and accuracy for staging of prostate cancer using ^11^C-Choline being 80%, 96% and 93%, respectively [5].

A high positive predictive value of ^11^C-Choline-PET for the detection of metastatic prostate carcinoma lesions has been shown, however also a low negative predictive value that depends on the limited capacity of ^11^C-Choline-PET to detect microscopic lesions [7]. However, ^11^C-Choline-PET was found to be superior to FDG-PET for re-staging prostatectomy cases with increasing serum PSA levels [8]. The radioactivity concentration or SUV of ^11^C-choline in prostate cancer and metastatic sites is mostly higher than three, while the SUV of FDG is considerably lower [6]. Furthermore, animal models show higher FDG uptake in inflammation compared to ^11^C-Choline-PET. High FDG-uptake is expected when transport across the cell membrane and metabolism through hexokinase activity are increased, which can be seen in tumours as well as with tissue inflammation. In contrast, ^11^C-Choline uptake in tumors is regulated by cell membrane synthesis, and is proportional to tumor cell proliferation. This is suggesting the feasibility of ^11^C-Choline-PET imaging for the differential diagnosis of cancer and chronic inflammation [9].

## Conclusions

In the case presented here, the combination op elevated uptake of FDG but not of ^11^C-Choline-PET suggested that the plexopathy was due to neuritis and not to tumor deposition. In our patient, it was important to detect possible prostate carcinoma metastasis causing plexopathy, which would be an indication for radiation therapy. As ^11^C-Choline PET did not detect neoplastic tissue, there was no rationale for radiation therapy of the sacral plexus.

## Abbreviations

FDG: fluorodeoxyglucose; EMG: electromyography; MRI: magnetic resonance imaging; PET: positron emission tomography; PSA: prostate specific antigen; SUV: standardized uptake value.

## Consent

Written informed consent was obtained from the patient for publication of this case report and accompanying images. A copy of the written consent is available for review by the Editor-in-Chief of this journal.

## Competing interests

The authors declare that they have no competing interests.

## Authors' contributions

ALB, CJZ, RHE and RS authors were fully involved with the management of this case while in hospital and have equally contributed to the design, drafting, and editing of the manuscript submitted.
